# Characteristics of *Chryseobacterium* bacteremia, associated risk factors and their antibiotic susceptibility pattern at a university hospital: a descriptive, retrospective study

**DOI:** 10.1099/acmi.0.000594.v3

**Published:** 2023-11-29

**Authors:** Radhika Chaudhary, Mitra Kar, Ashima Jamwal, Akanksha Dubey, Romya Singh, Nidhi Tejan, Chinmoy Sahu, Sangram Singh Patel, Pooja Kumari, Malay Ghar

**Affiliations:** ^1^​ Department of Microbiology, Sanjay Gandhi Postgraduate Institute of Medical Sciences, Lucknow, Uttar Pradesh-226014, India

**Keywords:** antimicrobial susceptibility profile, *Chryseobacterium *spp., immunocompromised, indwelling medical devices

## Abstract

**Introduction.:**

*
Chryseobacterium* species are emerging bacteria capable of causing nosocomial infections in immunocompromised patients or patients with indwelling medical devices.

**Hypothesis/ Gap statement.:**

Information about the incidence of *Chryseobacterium* bacteremia from worldwide literature is limited.

**Aim.:**

We aimed to recognize the clinical characteristics, frequency of distribution of different *Chryseobacterium* species isolates, and their antimicrobial susceptibility profile from bloodstream infections.

**Methods.:**

We performed a retrospective cohort study to identify all isolates of *Chryseobacterium* species from bloodstream infection from January 2018 to November 2022 at a university hospital in North India.

**Results.:**

We identified 42 non-duplicate isolates of *Chryseobacterium* species from bloodstream infection in the duration of our study. Mean age of the patients was 48.35±16.63 years. Men (22/42, 52.2 %) were more commonly affected in comparison to women (20/42, 47.6 %) but the difference was not significant. The most common species identified was *C. indologenes* (40/42, 95.24 %) followed by *C. gleum* (2/42, 4.76 %). The co-morbidities commonly encountered in our study were chronic kidney disease (21/42, 50.0 %) followed by diabetes mellitus (12/42, 28.6 %) and chronic obstructive pulmonary disease (8/42, 19.05 %). All patients had intravenous access to medications or fluid management via a central or peripheral line and mechanical ventilation was observed in 39 (39/42, 92.86 %) patients. All the isolates were susceptible to minocycline (100 %), followed by doxycycline (97.6 %) and trimethoprim-sulfamethoxazole (95.2 %).

**Conclusion.:**

*
Chryseobacterium* species are capable of causing pneumonia, bacteremia and urinary tract infection in immunocompromised patients. Early diagnosis and prompt treatment with appropriate antibiotics can prevent progression to septicemia.

## Data Summary

No supporting data in publicly available repositories (where available and appropriate) has been added to the main manuscript.

## Introduction

*Chryseobacterium* spp. are ubiquitous and are found mainly in water and soil [1]. In the hospital environment, these bacteria colonize wet and humid surfaces like water tanks, sinks, and washbasins acting as a source of infection [2]. Infections due to *Chryseobacterium* spp. are usually nosocomial and patients acquire infection through colonized medical devices coming in contact with fluids, e.g. respirators, humidifiers, syringes, and contaminated surgically implanted prosthetic devices [3–5]. *Chryseobacterium* spp. rarely causes infection in the immunocompetent host. Among immunocompromised patients, *Chryseobacterium* spp. have been implicated in patients of all age groups. These have been isolated from cases of meningitis, pneumonia, catheter-related bloodstream infections, endocarditis, biliary tract infections, ocular infections, skin and soft tissue infections, peritonitis, urinary tract infections, and surgical wound infection [6–9].

*Chryseobacterium* spp. belong to the family *Flavobacteriaceae* and was previously known as *Flavobacterium* CDC group IIb. It was first isolated as an opportunistic pathogen from the respiratory secretions of a patient suffering from ventilator-associated pneumonia (VAP) [10]. The commonly isolated species from clinical samples include *C. odoratum, C. meningosepticum, C. breve, C. multivorum* and *C. gleum, and C. indologenes*. Only *C. indologenes, C. gleum*, and *C. hominis* are known to cause infection in humans. *Chryseobacterium* spp. are Gram-negative, non-lactose fermenting, aerobic, non-motile bacilli producing oxidase and catalase enzymes. This bacterium also produces a distinct yellow-to-orange pigment on blood agar [11].

These bacteria can produce biofilms on foreign medical devices like catheters, and prosthetic implants, and protease production may be the virulence factor involved in the pathogenesis [12].

Nowadays with the increased incidence of carbapenem-resistant and extended-spectrum beta lactamase pathogens like *Escherichia coli, Acinetobacter baumannii*, and *Klebsiella pneumoniae* has led to indiscriminate use of drugs of last resort like tigecycline and colistin. This indiscriminate use of antimicrobials of last resort has led to *Chryseobacterium* species emerging as a significant problem in the critical healthcare setting [13, 14].

Intrinsic resistance to first, second, and third generation cephalosporins and carbapenems by virtue of production of molecular class A β-lactamase *bla_CIA_* and class B metallo-betalactamase *bla_IND_* is observed in *Chryseobacterium indologenes* [15, 16]. These exhibit resistance to other classes of antibiotics also which include aminoglycosides, chloramphenicol, clindamycin, tetracyclines, macrolide and teicoplanin, while piperacillin-tazobactam, minocycline, ceftazidime, trimethoprim-sulfamethoxazole, quinolones and rifampicin usually remain effective against this microorganism [2].

Data on antimicrobial susceptibility of *Chryseobacterium* spp. remains limited owing to its rare isolation from clinical specimens. No specific guidelines for antimicrobial susceptibility testing from CLSI (Clinical Laboratory Standards Institute) or EUCAST (European Committee for Antimicrobial Susceptibility Testing) have been defined for the genus *Chryseobacterium* spp.

There is limited data available on infections due to *Chryseobacterium* spp. and their antimicrobial susceptibility profile from across the world. Hence, we conducted this study to recognize the clinical characteristics, risk factors and drug susceptibility pattern of various *Chryseobacterium* spp. isolated from blood culture samples.

## Methods

### Study design and study population

This is a retrospective observational study where automated blood cultures that grew *Chryseobacterium* spp. (while no isolate with the identification of *Flavobacterium* genus was isolated in the study) were assessed for patient characteristics and antibiotic susceptibility patterns from January 2018 to November 2022 in the microbiology laboratory of a university hospital. The study was approved by the Institutional Ethics Committee (2021–48-EMP-EXP dated 29 November 2021). Informed consent was waived given our study is retrospective.

### Data collection

A set of automated BACTEC blood culture bottles (Becton Dickinson Diagnostic Instrument Systems, Sparks, MD) were received in the Bacteriology section of the Department of Microbiology and underwent standard microbiological processing like culture followed by microscopy and bacterial isolation. Further, the blood cultures exhibiting suspected growth of *Chryseobacterium* spp. were identified by a phenotypic method involving the addition of a drop of 10 % potassium hydroxide (KOH) over the colonies leading to change of colour from yellow to red indicating the presence of flexirubin pigment [17]. The identification was confirmed using matrix-assisted laser desorption ionization-time of flight mass spectrometry (MALDI-TOF MS) (BioMérieux, United States) in this study. Laboratory data of all the patients was retrospectively collected over the period of 5 years from the laboratory registers and clinical data was retrieved from the hospital information system (HIS) and patient files.

### Inclusion criteria

A true pathogen was defined depending on the time of positivity of blood culture and clinical and laboratory indexes in the study. Positive blood culture from samples collected before 48 h of admission were labelled as community-acquired bloodstream infection (BSI) while the positive blood culture collected after 48 h of admission were labelled as nosocomial in origin.

### Exclusion criteria

Isolates collected only on a single positive culture without any significant clinical parameters were considered a contaminant and excluded from the study.

### Antimicrobial susceptibility testing (AST)

The conventional (Kirby-Bauer disc diffusion method on Müller Hinton agar) method of AST was used in the study. As there are no defined guidelines for reporting Antimicrobial Susceptibility Testing (AST) for *Chryseobacterium* spp., we performed AST by Kirby-Bauer disc diffusion method on Müller Hinton agar using the antibiotic discs of amikacin (30 mcg), aztreonam (30 mcg), ceftazidime (30 mcg), cefoperazone-sulbactam (75/30 mcg), imipenem (10 mcg), meropenem (30 mcg), piperacillin-tazobactam (100/10 mcg), levofloxacin (5 mcg), ticarcillin-clavulanic acid (75/10 mcg), doxycycline (30 mcg), trimethoprim-sulfamethoxazole (1.25/23.75 mcg) and minocycline (30 mcg). The disc inhibition zone diameter determined by the Kirby-Bauer disc diffusion method and/ or MIC value determined by using the Epsilometeric-tests, which were interpreted in the laboratory using the susceptibility break points following the recommendation by the Clinical and Laboratory Standards Institute for non-Enterobacteriaceae and *Pseudomonas aeruginosa* [12].

### Treatment administered

We considered the use of appropriate antibiotics even if one drug from the panel was used in treatment of the patient.

### Statistical analysis

Categorical variables were described using percentages and a Chi-square test was applied. Univariate analysis of the risk factors in all patients included in our study was assessed using Kaplan-Meier survival analysis. All statistical analyses were performed using SPSS statistical software (IBM SPSS version 20, Armonk, N.Y.).

## Results

**Fig. 1.**
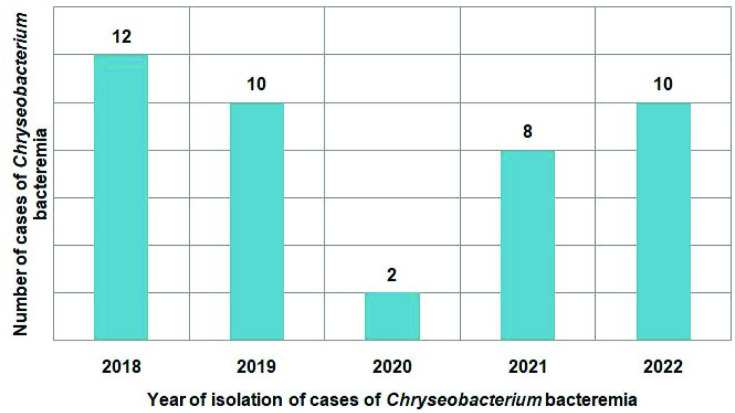
The prevalence of the 42 cases of *Chryseobacterium* bacteremia by year during the 5 year study period (*N*=42).

In our study, a total of 42 isolates *Chryseobacterium indologenes *, *n*=40 (40/42, 95.24 %); and *Chryseobacterium gleum *, *n*=2 (2/42, 4.76 %) were identified from bloodstream infections during the course of the 5 year study period. Fig. 1 represents the prevalence of these 42 cases of *Chryseobacterium* bacteremia by year during the 5 year study period. All the isolates identified in this study were subjected to identification by use of Matrix assisted laser desorption/ ionization-time of flight mass spectrometry (MALDI-TOF MS) and all 42 isolates were included in the study after confirmatory identification using MALDI-TOF MS with a confidence level of 99.9 %.

Mean age of the patients in our study cohort was 48.35±16.63 years. Men (22/42, 52.2 %) were more commonly affected than women (20/42, 47.6 %) but this difference was not significant. The majority of these patients were admitted to critical care medicine 66.6 % (28/42) followed by anaesthesia 19 % (8/42), emergency 7.1 % (3/42) and other departments including nephrology, cardiology and pulmonary medicine. Forty (40/42, 95.24 %) cases of bacteremia were nosocomially acquired while only two (2/42, 4.76 %) cases were community-acquired. Acute or chronic kidney disease was observed in nine (9/42, 21.43 %) patients followed by seven (7/42, 16.67 %) patients with acute necrotizing pancreatitis and five (5/42, 11.90 %) patients with severe dengue. Other underlying comorbidities include gastrointestinal diseases like intestinal obstruction, Gall bladder perforation following cholecystectomy, and angiodysplasia of the colon in three (3/42, 7.14 %) patients and cardiovascular diseases like valve regurgitation, myocarditis, infective endocarditis, and coronary artery disease in four (4/42, 9.52 %) patients. Indwelling devices were identified as significant predisposing factors attributing to *Chryseobacterium* bacteremia. All the patients (42/42, 100 %) admitted to our tertiary care centre were managed on intravenous access for medication and fluid management using central or peripheral line and 73.81 % (31/42, 73.81 %) were managed on mechanical ventilation (Table 1).

**Table 1.** Comparison of demographic and clinical characteristics among the *Chryseobacterium* bacteremia patients who died or survived (*n*=42)

Variables	Survived (*n*=19)	Dead (*n*=23)	*p*-value
**Age (mean±SD) in years**	48.82±19.30	48.00±14.79	0.877
**Gender (male, n%)**	11 (57.89 %)	11 (47.83 %)	0.516
**Underlying comorbidities**			
Kidney disease	9 (47.37 %)	13 (56.52 %)	0.554
Acute necrotizing pancreatitis	0 (0.0 %)	7 (30.43 %)	0.008*
Hypertension	3 (15.79 %)	10 (43.48 %)	0.050*
Diabetes mellitus	2 (10.53 %)	10 (43.48 %)	0.019*
Respiratory diseases	3 (15.79 %)	7 (30.43 %)	0.267
Cardiovascular diseases	0 (0.0 %)	4 (17.39 %)	0.050*
Gastrointestinal diseases	1 (5.26 %)	2 (8.69 %)	0.667
History of immunosuppressive drugs	3 (15.79 %)	2 (8.69 %)	0.480
History of tuberculosis	0 (0.0 %)	4 (17.39 %)	0.050*
**Presence of indwelling devices**			
Central venous catheter	11 (57.89 %)	22 (95.65 %)	0.003*
Mechanical ventilator	9 (47.37 %)	22 (95.65 %)	<0.001*
Hemodialysis	6 (31.58 %)	8 (34.78 %)	0.826
**Diagnostic parameters**			
Procalcitonin (in ng ml^−1^)	0.31±0.30	31.36±33.43	<0.001*
Total leucocyte count (cells mm^−3^)	13947.37±8024.37	21030.44±8042.41	0.007*
**Other parameters**			
Length of hospital stay (in days)	19.89±24.93	43.22±26.91	0.006*
Nosocomially acquired bacteremia	9 (47.37 %)	23 (100.0 %)	<0.001*
Community acquired bacteremia	10 (52.63 %)	0 (0.0 %)	<0.001*
Patients with co-infections	11 (57.89 %)	19 (82.61 %)	0.078
Clinically diagnosed septicemia	7 (36.84 %)	19 (82.61 %)	0.002*
Appropriate antibiotics administered	5 (26.32 %)	7 (30.43 %)	0.002*

*p-value ≤ 0.5 is statistically significant.

Co-infections with other bacterial and fungal agents were observed in 30 (30/42, 71.43 %) patients. The co-infection most commonly encountered was with *Candida* spp. (10/30, 33.33 %) followed by five (5/30, 16.67 %) cases of *A. baumannii *, five (5/30, 16.67 %) cases of *Klebsiella pneumoniae *, four (4/30, 13.33 %) cases of *Pseudomonas aeruginosa* and six (6/30, 20.0 %) cases of *Escherichia coli *.

Broad spectrum antibiotics were started empirically for all patients with bloodstream infections. Meropenem was administered in 28 (28/42, 66.67 %) patients in our inpatient departments followed by fluoroquinolones (7/42, 16.67 %) and third generation cephalosporins (5/42, 11.90 %). All (42/42, 100 %) isolates were susceptible to minocycline while 97.62 and 95.24 % of isolates were susceptible to doxycycline and trimethoprim-sulfamethoxazole respectively. However, the susceptibility to levofloxacin, piperacillin-tazobactam, cefoperazone-sulbactam, ceftazidime, amikacin, and ticarcillin-clavulanic acid were 42.86, 30.95, 26.19, 11.90, 9.52 and 9.52% respectively. None of the isolates were reported with an intermediate pattern of susceptibility to the antibiotics. The susceptibility of isolates to carbapenems (imipenem and meropenem) was only 4.7 % representing an increasing resistance to carbapenems which could be attributed to the rampant use of carbapenems in empirical therapy of patients in our hospital (Table 2). Appropriate antibiotics administered in accordance with the drug susceptibility pattern was observed in only 12 (12/42, 28.57 %) patients.

**Table 2.** Antimicrobial susceptibility profile of *Chryseobacterium* spp. isolated from bloodstream infections of patients included in our study cohort (*n*=42)

Antibiotics tested for susceptibility	Susceptible	Intermediate	Resistant
**Amikacin**	4 (9.52 %)	0 (0.0 %)	38 (90.48 %)
**Ceftazidime**	5 (11.90 %)	0 (0.0 %)	37 (88.10 %)
**Cefoperazone - sulbactam**	11 (26.19 %)	0 (0.0 %)	31 (73.81 %)
**Doxycycline**	41 (97.62 %)	0 (0.0 %)	1 (2.38 %)
**Imipenem**	2 (4.76 %)	0 (0.0 %)	40 (95.24 %)
**Levofloxacin**	18 (42.86 %)	0 (0.0 %)	24 (57.14 %)
**Meropenem**	2 (4.76 %)	0 (0.0 %)	40 (95.24 %)
**Minocycline**	42 (100.0 %)	0 (0.0 %)	0 (0.0 %)
**Piperacillin – tazobactam**	13 (30.95 %)	0 (0.0 %)	29 (69.05 %)
**Ticarcillin – clavulanic acid**	4 (9.52 %)	0 (0.0 %)	38 (90.48 %)
**Trimethoprim - sulphamethoxazole**	40 (95.24 %)	0 (0.0 %)	2 (4.76 %)

The mode of administration of all antibiotics to the patients included in this study was by intravenous route to achieve adequate antibiotic concentration in the bloodstream and counteract the life-threatening bacteremia. Keeping in mind the immunocompromised state of the patients in this study it was the most feasible method of drug administration to improve patient outcome. As observed in the electronic hospital records, the patients who survived were later shifted onto oral antibiotics on discharge.

The overall mortality rate recorded in our study was 54.76 % (23/42). We also compared the demographic and clinical characteristics with the survival in patients with *Chryseobacterium* bacteremia (Table 1). The underlying comorbidities significantly associated with death in our patient cohort were acute necrotizing pancreatitis, hypertension and diabetes mellitus. The presence of indwelling devices like central venous catheter (*P*=0.003) in 33 (33/42, 78.57 %) patients and mechanical ventilation (*P*<0.001) in 31 (31/42, 73.81 %) patients were statistically significant risk factors responsible for mortality. Procalcitonin (*P*<0.001) and total leucocyte counts (*P*=0.007) were used as a significant parameter for diagnosing septicemia, and higher values of procalcitonin and total leucocyte count were recorded in patients who died. An increased duration of hospitalization was significantly associated with increased deaths among our patient cohort. Cases of nosocomially acquired bacteremia were more commonly associated with death. Appropriate administration of antibiotics in patients who survived was statistically significant in comparison to those who succumbed to *Chryseobacterium* bacteremia. Only twelve (12/42, 28.57 %) patients of *Chryseobacterium* bacteremia showed antibiotic susceptibility to carbapenems, third-generation cephalosporins and extended spectrum beta-lactams that were being administered empirically, which could be attributed to the rampant use of carbapenems and other drugs for empirical treatment in the hospital, rendering them ineffective and increased association with indwelling devices, we recorded a high rate of mortality in our study cohort.

## Discussion

*Chryseobacterium* infections have been perceived in the extremes of age (premature infants and elderly), immunocompromised patients with chronic obstructive pulmonary disease, diabetes mellitus, chronic kidney disease, cardiovascular disease, malignancies and patients on broad-spectrum antibiotics [18]. In this study, common co-morbidities encountered were kidney diseases (22/42, 52.38 %) followed by diabetes mellitus (12/42, 28.57 %) and respiratory diseases (10/42, 23.81 %).

*Chryseobacterium* spp. are capable of inflicting a range of infections like bacteremia, pneumonia, urinary tract infection, and biliary tract infection. The repeated isolation of *Chryseobacterium* spp. from blood specimens was observed in all cases included in this study, signifying its clinical relevance as a bloodstream pathogen which corroborates the results of studies by Chen *et al.* [12] and Yadav *et al.* [19]. The predisposing risk factors are long-term indwelling devices like endotracheal tubes [20], central and peripheral lines [21] and urinary catheter [22], which was also experienced in this study given an statistically significant number of deaths in comparison to patients who survived with concomitant presence of *Chryseobacterium* bacteremia and indwelling catheters especially central venous catheters (33/42, 78.57 %) and / or mechanical ventilation (31/42, 73.81 %).

The majority of patients were hospitalized in the critical care unit with concomitant presence of indwelling catheters. All patients (42/42, 100 %) in our study cohort had a central and / or peripheral line for intravenous access and 31 (31/42, 73.81 %) were managed for respiratory distress on mechanical ventilator. Thirty-two (32/42, 76.19 %) cases of nosocomially acquired bacteremia and ten (10/42, 23.81 %) cases of community-acquired bacteremia were identified. This nosocomial acquisition of *Chryseobacterium* bacteremia can be attributed to its property to colonize and form biofilms on humid medical devices like a humidifier, respirators, contaminated catheters and surgical instruments and spread in a hospital setting through contaminated medical devices [18]. The above finding was consolidated by a study from North India that reported a history of indwelling medical devices in 55.56 % (10/18, 55.56 %) of their patients [19, 23, 24].

The *Chryseobacterium* spp. reported in our study were *Chryseobacterium indologenes* (40/42, 95.24 %) followed by *Chryseobacterium gleum* (2/42, 4.76 %)*,* which was correlates with a study by Singh *et al.* that also reported *C. indologenes* (18/20, 90.0 %) as the commonly isolated species followed by *C. gleum* (2/20, 10.0 %) from North India [18, 19, 25].

A study by Lin *et al.* illustrates the disparity in clinical characteristics and antimicrobial susceptibility patterns between *C. indologenes* and *C. gleum.* During their study period from 2005 to 2017, they identified and studied 84 isolates of *C. indologenes* and 42 isolates of *C. gleum* and associated *C. gleum* with more comorbidities, bacteremia, low case fatality rate and it was also deemed highly susceptible to several antimicrobial agents [18].

*Chryseobacterium* spp. are usually susceptible to minocycline, trimethoprim-sulfamethoxazole, quinolones, and rifampicin [22]. All isolates were susceptible to minocycline (100 %), while the isolates were found to be 97.6 and 95.2% susceptible to doxycycline and trimethoprim-sulfamethoxazole, respectively. However, the susceptibility of levofloxacin, piperacillin-tazobactam, cefoperazone-sulbactam, ceftazidime, amikacin, ticarcillin-clavulanic acid were 42.8, 30.9, 26.1, 11.9, 9.5 and 9.5% respectively. *Chryseobacterium* spp. showed 95.3 % resistance to carbapenem. Zhang *et al.* determined the AST of *Chryseobacterium* spp. using broth microdilution (BMD) and demonstrated that minocycline (98.5 %) and trimethoprim-sulfamethoxazole (97.8 %) were more potent antimicrobial agents which corresponds to the antibiotic susceptibility pattern observed in our study [22]. In a study conducted in the duration from 1992 to 1995, the isolates of *C. indologenes* were least susceptible to trimethoprim-sulfamethoxazole (30.6 %) followed by 41.7 % susceptibility to ceftazidime and most susceptible to minocycline (27/36, 75 %) [26].

According to Kirby *et al.*, more than 85 % of 20 *C. indologenes* isolates collected between 1997 to 2001 were susceptible to ceftazidime, cefepime, ciprofloxacin, levofloxacin, piperacillin, piperacillin-tazobactam and trimethoprim-sulfamethoxazole [2]. In a study by Chen *et al.,* 113 *C. indologenes* isolates showed a low susceptibility of 29.4 % to piperacillin-tazobactam followed by 31.6 % susceptibility to ciprofloxacin, 34.4 % susceptibility to levofloxacin, 41.8 % susceptibility to tigecycline and 87.6 % susceptibility to trimethoprim-sulfamethoxazole [12].

Lin *et al.* reported that 126 isolates of *Chryseobacterium* spp. collected between 2005–2017 showed 73 % susceptibility to minocycline followed by 47.6 % trimethoprim-sulfamethoxazole, 34.1 % tigecycline, 32.5 % levofloxacin, 19.8 % piperacillin-tazobactam, and 19 % piperacillin [18].

According to this study and previously published studies minocycline, trimethoprim-sulfamethoxazole and doxycycline were the most effective antibiotics in treatment of *Chryseobacterium* bacteremia. Among the patients who survived in this study, only five (5/19, 26.32 %) patients received appropriate antibiotics whereas seven (7/23, 30.43 %) patients who died had received appropriate antibiotics. On comparison of drug administration among the two groups, more adequate antibiotic administration was observed in the patients who died which could be attributed to the delayed administration of appropriate antibiotic therapy associated with adverse outcome as observed in a study by Falcone *et al.* [27].

Our study had certain limitations. Firstly, it is a single centre study with very few isolates that does not represent the picture of infections in the other hospitals in our geographical location. Secondly, it is a retrospective study where all the data was extracted from the patient records which could lead to investigator bias or information bias. Thirdly, we could not perform broth microdilution (BMD) method of antibiotic susceptibility testing on these isolates as all isolates were recorded retrospectively. The above limitation also includes a lack of standardization in interpretation of breakpoints for susceptibility testing (both disc diameter and Epsilometeric-test) for *Chryseobacterium *, in addition to not performing broth microdilution (BMD).

## Conclusion

To the best of our knowledge, this is the first study from northern India that reported clinical characteristics and an antimicrobial susceptibility profile of *Chryseobacterium* spp. With the advent of MALDI-TOF MS, early and accurate identification of these emerging microorganisms facilitates management of infection. Very limited data is available on the antimicrobial susceptibility pattern of *Chryseobacterium* spp., so there is a need to determine the susceptibility data of this microorganism worldwide to provide a uniform platform for performing antimicrobial susceptibility testing. Minocycline is a suitable antibiotic for *Chryseobacterium* bacteremia.
